# Antibiotic Selection and Duration for Catheter-Associated Urinary Tract Infection in Non-Hospitalized Older Adults: A Population-Based Cohort Study

**DOI:** 10.1017/ash.2023.176

**Published:** 2023-08-01

**Authors:** Bradley J. Langford, Nick Daneman, Christina Diong, Samantha M. Lee, Daniel J. Fridman, Jennie Johnstone, Derek MacFadden, Kwadwo Mponponsuo, Samir N. Patel, Kevin L. Schwartz, Kevin A. Brown

**Affiliations:** 1 Public Health Ontario, Toronto, Canada; 2 Dalla Lana School of Public Health, University of Toronto, Toronto, Canada; 3 Sunnybrook Health Sciences Centre, Toronto, Canada; 4 ICES, Toronto, Canada; 5 Institute of Health Policy Management and Evaluation, University of Toronto, Toronto, Canada; 6 Sinai Health, Toronto, Canada; 7 Department of Laboratory Medicine and Pathobiology, University of Toronto, Toronto, Canada; 8 Ottawa Hospital Research Institute, Ottawa, Ontario, Canada; 9 Department of Medicine Section of Infectious Diseases, University of Calgary, Calgary, Canada; 10 St. Joseph’s Health Centre, Unity Health, Toronto, Canada

## Abstract

**Background:**

We sought to evaluate the impact of antibiotic selection and duration of therapy on treatment failure in older adults with catheter-associated urinary tract infection (CA-UTI).

**Methods:**

We conducted a population-based cohort study comparing antibiotic treatment options and duration of therapy for non-hospitalized adults aged 66 and older with presumed CA-UTI (defined as an antibiotic prescription and an organism identified in urine culture in a patient with urinary catheterization documented within the prior 90 d). The primary outcome was treatment failure, a composite of repeat urinary antibiotic prescribing, positive blood culture with the same organism, all-cause hospitalization or mortality, within 60 days. We determined the risk of treatment failure accounting for age, sex, comorbidities, and healthcare exposure using log-binomial regression.

**Results:**

Of 4,436 CA-UTI patients, 2,709 (61.1%) experienced treatment failure. Compared to a reference of TMP-SMX (61.9% failure), of those treated with fluoroquinolones, 56.3% experienced failure (RR 0.91, 95% CI: 0.85–0.98) and 60.9% of patients treated with nitrofurantoin experienced failure (RR 1.02, 95% CI: 0.94–1.10). Compared to 5–7 days of therapy (treatment failure: 59.4%), 1–4 days was associated with 69.5% failure (RR 1.15, 95% CI: 1.05–1.27), and 8–14 days was associated with a 62.0% failure (RR 1.05, 95% CI: 0.99–1.11).

**Conclusions:**

Although most treatment options for CA-UTI have a similar risk of treatment failure, fluoroquinolones, and treatment durations ≥ 5 days in duration appear to be associated with modestly improved clinical outcomes. From a duration of therapy perspective, this study provides reassurance that relatively short courses of 5–7 days may be reasonable for CA-UTI.

## Background

Catheter-associated urinary tract infection (CA-UTI) is the most common healthcare-acquired infection. Indwelling urethral catheters are frequently used for patients in healthcare settings.^1^ Chronic urinary catheter use is common in long-term care residents with a prevalence of 5%–15%.^2^ CA-UTI is particularly burdensome in older long-term care residents given the high risk of bacteremia with a urinary organism and the increased risk of adverse events due to antibiotic therapy.^3,4^ Beyond the negative impact on individual patient outcomes associated with CA-UTI, catheterized patients frequently receive antimicrobial treatment and as such may act as reservoirs of antimicrobial resistance and a source of outbreaks, affecting the broader population.^1^

Reduction in unnecessary catheter use and appropriate urine culturing practices are important antimicrobial stewardship initiatives to prevent CA-UTI and unnecessary treatment for catheter-associated asymptomatic bacteriuria.^5–8^ However, there remain opportunities to optimize antimicrobial use in patients with established CA-UTI. There is a lack of data to support optimal antibiotic selection in patients with CA-UTI. For example, while Infectious Diseases Society of America CA-UTI guidelines do not specifically recommend nitrofurantoin as treatment for CA-UTI, the United Kingdom’s National Institute for Health and Care Excellence guidelines recommend this agent as first-line therapy.^9,10^ Similarly, although guidelines commonly recommend a minimum of 7 days of antibiotic therapy for CA-UTI,^9,10^ there are limited high-quality data to support this recommendation. However, individual studies suggest that durations as short as 3 to 5 days may be reasonable.^11–13^ Additional evidence in support of narrower spectrum therapy and shorter courses of therapy can help advance antimicrobial stewardship efforts for these patients.

We sought to evaluate the impact of antibiotic selection and duration of therapy on treatment failure in older non-hospitalized adults with CA-UTI.

## Methods

### Study Design

We conducted a population-based retrospective cohort study comparing antibiotic treatment options and duration of therapy for non-hospitalized patients aged 66 and older with CA-UTI using administrative data.

### Setting

This study took place in Ontario, Canada’s most populous province (15.1 million in 2022). Health care is provided in Ontario via a universal single-payer health insurance model.

### Data Sources

We obtained study data from linked population-wide administrative datasets housed at ICES (formerly known as the Institute for Clinical Evaluative Sciences). ICES is an independent, non-profit research institute whose legal status under Ontario’s health information privacy law allows it to collect and analyze healthcare and demographic data, without consent, for health system evaluation and improvement.

Urine culture and susceptibility data were collected from the Ontario Laboratories Information System (OLIS), which is a province-wide repository for laboratory results.^14^ We collected antibiotic use data from the Ontario Drug Benefit (ODB) database, which includes outpatient medication dispensing data for adults over 65 years. Previous validation of ODB has shown 99.3% accuracy compared with chart abstraction of the actual prescription.^15^ Demographics and patient outcomes data were collected from the Registered Persons Database, the Ontario Health Insurance Plan (OHIP) Database, and Canadian Institute for Health Information (CIHI) National Ambulatory Care Recording System, CIHI Discharge Abstract Database (DAD), and Continuing Care Reporting System-Long-Term Care (CCRS-LTC). These datasets are linked using unique encoded identifiers and analyzed at ICES and have been widely used for studies of antimicrobial harms and benefits.^16,17^

### Patient Selection Criteria

Adults aged 66 years (aged 65 yr plus 1 yr to ensure an adequate look back) and older residing in the community or long-term-care setting were eligible for inclusion. Those hospitalized within the prior 30 days, with evidence of complicated or upper UTI, including pyelonephritis, renal and perinephric urethral abscess, prostatitis, acute and chronic prostatitis, orchitis/epididymitis (see codes in supplement) or a positive blood culture within 3 days prior to the index date, were excluded. In order to identify patients that were likely catheterized at the time of the index UTI with a high sensitivity, we included anyone with a catheterization code (codes provided in supplement) within 90 days prior to an index antibiotic prescription. Patients with (1) a first positive urine culture collected during the period from 1 January 2016 to 31 December 2020 (2) prescribed an eligible antibiotic either empiric or targeted treatment to which the culture was susceptible or not reported and (3) within +/– 3 days of urine culture finalization were included in the cohort.

### Exposure

Eligible antibiotics included amoxicillin, amoxicillin-clavulanate, cephalexin, cefadroxil, nitrofurantoin, trimethoprim-sulfamethoxazole (TMP-SMX), ciprofloxacin, levofloxacin, and fosfomycin prescribed for 1–14 days in duration. The first day of dispensation was considered the index date. Patients were excluded if more than one type of antibiotic was prescribed within 1 day of the index prescription. Since some long-term care patients receive scheduled dispensing (eg, weekly), consecutive prescriptions for the same antibiotic for 7 days were combined into a single course if the end date was within 1 days of the start date of the subsequent prescription.

### Outcome

The primary outcome was treatment failure which was a composite of repeat urinary antibiotic prescribing (from antibiotics listed above), all-cause hospitalization, positive blood culture with the same organism, or all-cause mortality, within 60 days of index prescription. Although less specific, all-cause hospitalization and mortality ensure that patient important, objective, and clinically meaningful events are included as part of the primary outcome, and such metrics have been used to evaluate antimicrobial stewardship initiatives.^18,19^ Secondary outcomes included components of the primary outcome as well as positive urine culture with the same organism, and cause-specific hospitalization or ED visit for pyelonephritis, sepsis, or bloodstream infection, within 60 days of index prescription. Antibiotic harms were evaluated using a composite outcome comprised of *C. difficile*, antibiotic allergy, diarrhea, and general medication adverse events.

### Patient Covariates

Several patient characteristics were collected including age, sex, geographical region, comorbidities, healthcare exposure, time from catheterization to index prescription, time from culture finalization to index prescription, organism category (*E. coli* vs non-*E.coli*), number of cultures, and number of antibiotics in previous year.

### Statistical Analysis

We described the CA-UTI cohort descriptively, with medians and interquartile range (IQR) where appropriate. The relationship between antibiotic selection, antibiotic duration, and treatment failure was evaluated in a log-binomial regression model with antibiotics grouped by class, and antibiotic duration grouped into 1–4, 5–7, 8–14-day courses. We developed an unadjusted (including only selection and duration variables) and adjusted model (also including age, sex, Charlson comorbidity score, acute care, and long-term care days in the past 12 mo). We also performed a stratified analysis to evaluate any differences between patients residing in community versus LTC settings. Risk of treatment failure was compared between treatment regimen options and expressed as a risk ratio (RR) with 95% confidence intervals (CI). All analyses were conducted in SAS version 9.4 (Cary, NC).

### Sensitivity Analyses

We performed two sensitivity analyses to test the robustness of our findings. Firstly, to ensure the treatment failure outcome was not driven by early changes due to culture and susceptibility results, we excluded any patient in which antibiotics were altered within +/– 3 days of culture finalization. A second sensitivity analysis used a threshold of 30 days, rather than 90 days, to look back for catheterization to increase the specificity and likelihood patients were catheterized at the time of the index prescription.

## Results

### Patients with CA-UTI

**Figure 1.**
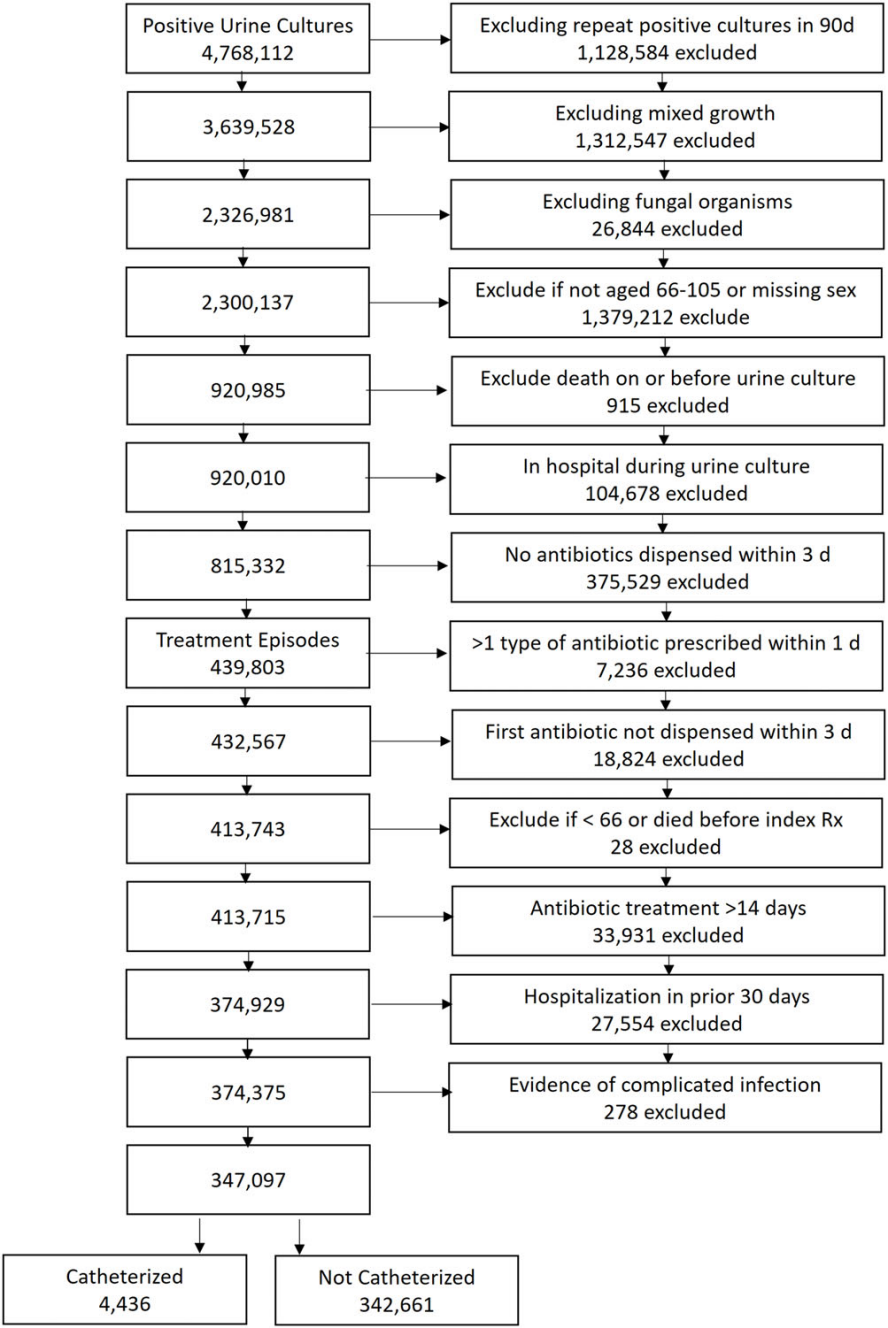
Cohort flow diagram

During the study period, after exclusions, there were 347,097 eligible older Ontario residents with positive urine cultures receiving antibiotics to treat a presumed urinary tract infection. Of these patients, 4,436 had documented urinary catheterization in the past 90 days and were considered presumed CA-UTI (Figure 1). The median time from catheterization to index antibiotic prescription was 35 days (IQR 11 to 60 d). *E. coli* was the causative organism in 33.7% (n = 1,493) of patients.

The median age of CA-UTI patients was 81 (IQR 74 to 88) and 62.6% (n = 2,777) were male. The most common Charlson comorbidity score was 0 (34.9%, n = 1,549). Median days spent in acute care in the past 12 months was 1 (IQR 0 to 11) and 38.9% (n = 1727) were long-term care residents. Fluoroquinolones were most commonly prescribed (30.7%, n = 1360), followed by nitrofurantoin (24.4%, n = 1083), and TMP-SMX (15.3%, n = 678). Patient characteristics varied by antibiotic selection. For example, patients receiving nitrofurantoin were slightly younger (median age 79, IQR 72 to 86) and a greater proportion were female (52.2%, n = 565) than those receiving other agents (Table 1).

**Table 1.** Characteristics of CA-UTI Patients Based on Antibiotic Received

Patient Characteristic		Total	Amoxicillin Ampicillin	Amoxicillin-clavulanate	Cefadroxil, Cephalexin	Nitrofurantoin	Trimethoprim-sulfamethoxazole	Ciprofloxacin, Levofloxacin	Fosfomycin
		N = 4,436	N = 306	N = 300	N = 431	N = 1,083	N = 678	N = 1,360	N = 278
Age (years)	Mean (SD)	81.1 (8.6)	82.8 (8.6)	82.6 (8.8)	82.5 (8.5)	79.4 (8.4)	81.0 (8.4)	81.2 (8.5)	81.4 (8.6)
	Median (Q1-Q3)	81 (74–88)	83 (76–89)	84 (75–90)	83 (76–89)	79 (72–86)	81 (74–88)	81 (74–88)	82 (74–88)
Age group	66–74	1,204 (27.1%)	61 (19.9%)	70 (23.3%)	92 (21.3%)	368 (34.0%)	179 (26.4%)	361 (26.5%)	73 (26.3%)
	75–84	1,520 (34.3%)	103 (33.7%)	85 (28.3%)	139 (32.3%)	380 (35.1%)	246 (36.3%)	475 (34.9%)	92 (33.1%)
	85+	1,712 (38.6%)	142 (46.4%)	145 (48.3%)	200 (46.4%)	335 (30.9%)	253 (37.3%)	524 (38.5%)	113 (40.6%)
Sex	Female	1,659 (37.4%)	114 (37.3%)	90 (30.0%)	155 (36.0%)	565 (52.2%)	202 (29.8%)	360 (26.5%)	173 (62.2%)
	Male	2,777 (62.6%)	192 (62.7%)	210 (70.0%)	276 (64.0%)	518 (47.8%)	476 (70.2%)	1,000 (73.5%)	105 (37.8%)
Charlson comorbidity index	0	1,549 (34.9%)	102 (33.3%)	94 (31.3%)	142 (32.9%)	384 (35.5%)	248 (36.6%)	500 (36.8%)	79 (28.4%)
	1	647 (14.6%)	50 (16.3%)	43 (14.3%)	78 (18.1%)	146 (13.5%)	103 (15.2%)	186 (13.7%)	41 (14.7%)
	2+	1,424 (32.1%)	105 (34.3%)	111 (37.0%)	137 (31.8%)	312 (28.8%)	200 (29.5%)	451 (33.2%)	108 (38.8%)
	No hospitalization	816 (18.4%)	49 (16.0%)	52 (17.3%)	74 (17.2%)	241 (22.3%)	127 (18.7%)	223 (16.4%)	50 (18.0%)
Days spent in acute care hospital prior to study	Mean (SD)	11.3 (26.1)	15.5 (32.3)	11.4 (25.3)	11.7 (24.1)	10.9 (25.4)	11.4 (28.4)	10.1 (24.3)	13.2 (27.1)
	Median (Q1-Q3)	1 (0–11)	4 (0–16)	2 (0–14)	1 (0–13)	0 (0–10)	1 (0–10)	1 (0–10)	2 (0–14)
Days spent in LTC	Mean (SD)	94.2 (149.5)	135.2 (165.4)	152.2 (164.0)	112.1 (157.1)	75.1 (139.7)	110.5 (155.7)	66.4 (131.2)	129.0 (165.9)
Number of primary care visits in previous year	Median (Q1-Q3)	0 (0–191)	0 (0–354)	68 (0–357)	0 (0–326)	0 (0–49)	0 (0–299)	0 (0–24)	0 (0–343)
	Mean (SD)	5.9 (6.4)	5.3 (6.5)	5.0 (5.9)	5.7 (6.3)	6.3 (6.4)	5.2 (5.5)	6.2 (6.4)	6.6 (8.8)
	Median (Q1-Q3)	4 (1–9)	3 (1–8)	3 (0–7)	4 (1–9)	5 (2–9)	4 (1–8)	5 (2–9)	4 (1–9)
Time from catheterization to index prescription (day)	Mean (SD)	37.2 (27.6)	42.2 (27.0)	40.4 (28.3)	37.1 (27.5)	34.4 (27.1)	38.4 (27.2)	35.8 (27.9)	42.2 (27.5)
	Median (Q1-Q3)	35 (11–60)	41 (18–63)	40 (14–65)	32 (11–60)	31 (8–57)	36 (14–60)	32 (10–59)	41 (16–65)
Antibiotic time relative to culture finalization	Before	524 (11.8%)	16 (5.2%)	48 (16.0%)	54 (12.5%)	119 (11.0%)	86 (12.7%)	150 (11.0%)	51 (18.3%)
	After	3,912 (88.2%)	290 (94.8%)	252 (84.0%)	377 (87.5%)	964 (89.0%)	592 (87.3%)	1,210 (89.0%)	227 (81.7%)
Organism	Non-*E. coli*	2,943 (66.3%)	233 (76.1%)	198 (66.0%)	277 (64.3%)	573 (52.9%)	450 (66.4%)	1,068 (78.5%)	144 (51.8%)
	*E. coli*	1,493 (33.7%)	73 (23.9%)	102 (34.0%)	154 (35.7%)	510 (47.1%)	228 (33.6%)	292 (21.5%)	134 (48.2%)
Number of urine culture in previous year	Mean (SD)	2.9 (3.3)	3.3 (3.6)	3.6 (4.1)	2.7 (2.7)	3.0 (3.5)	2.9 (3.2)	2.5 (2.6)	4.3 (5.0)
	Median (Q1-Q3)	2 (1–4)	2 (1–5)	2 (1–5)	2 (1–4)	2 (1–4)	2 (1–4)	2 (1–4)	3 (1–5)
Number of positive urine culture in previous year	Mean (SD)	1.2 (1.9)	1.4 (2.2)	1.6 (2.2)	1.1 (1.6)	1.2 (2.0)	1.2 (1.8)	0.9 (1.6)	1.8 (2.8)
	Median (Q1-Q3)	0 (0–2)	1 (0–2)	1 (0–3)	0 (0–2)	0 (0–2)	0 (0–2)	0 (0–1)	1 (0–3)
Number of antibiotic courses in previous year	Mean (SD)	4.1 (6.0)	4.0 (5.0)	5.3 (6.5)	4.2 (6.4)	4.4 (7.0)	4.3 (6.3)	3.3 (4.3)	5.5 (7.8)
	Median (Q1-Q3)	2 (1–5)	3 (1–5)	4 (2–7)	2 (1–5)	2 (1–5)	3 (1–5)	2 (1–5)	3 (1–6)

The most common prescribed duration was 7 days (54.6%, n = 2,424) followed by 8–10 days (17.1%, n = 759), then 5–6 days (13.0%, n = 579). Male patients received longer courses of antibiotic therapy. For example, those receiving 11–14 days were predominantly male (79.2%, n = 152) (Table 2).

**Table 2.** Characteristics of CA-UTI Patients Based on Antibiotic Duration Prescribed

			Antibiotic duration					
		Total	1–2 d	3–4 d	5–6 d	7 d	8–10 d	11–14 d
Patient Characteristic		N = 4,436	N = 310	N = 172	N = 579	N = 2,424	N = 759	N = 192
Age (years)	Mean (SD)	81.1 (8.6)	80.3 (8.5)	80.8 (9.1)	81.5 (8.5)	81.2 (8.6)	80.9 (8.4)	80.0 (8.8)
	Median (Q1–Q3)	81 (74–88)	80 (73–87)	82 (73–89)	82 (75–88)	81 (74–88)	81 (74–88)	80 (73–88)
Age group	1. 66–74	1,204 (27.1%)	93 (30.0%)	58 (33.7%)	139 (24.0%)	645 (26.6%)	205 (27.0%)	64 (33.3%)
	2. 75–84	1,520 (34.3%)	104 (33.5%)	44 (25.6%)	211 (36.4%)	833 (34.4%)	269 (35.4%)	59 (30.7%)
	3. 85+	1,712 (38.6%)	113 (36.5%)	70 (40.7%)	229 (39.6%)	946 (39.0%)	285 (37.5%)	69 (35.9%)
Sex	Female	1,659 (37.4%)	218 (70.3%)	73 (42.4%)	259 (44.7%)	854 (35.2%)	215 (28.3%)	40 (20.8%)
	Male	2,777 (62.6%)	92 (29.7%)	99 (57.6%)	320 (55.3%)	1,570 (64.8%)	544 (71.7%)	152 (79.2%)
Charlson comorbidity index	0	1,549 (34.9%)	98 (31.6%)	59 (34.3%)	205 (35.4%)	834 (34.4%)	281 (37.0%)	72 (37.5%)
	1	647 (14.6%)	38 (12.3%)	29 (16.9%)	87 (15.0%)	358 (14.8%)	108 (14.2%)	27 (14.1%)
	2+	1,424 (32.1%)	103 (33.2%)	52 (30.2%)	174 (30.1%)	800 (33.0%)	241 (31.8%)	54 (28.1%)
	No hospitalization	816 (18.4%)	71 (22.9%)	32 (18.6%)	113 (19.5%)	432 (17.8%)	129 (17.0%)	39 (20.3%)
Days spent in acute care hospital	Mean (SD)	11.3 (26.1)	10.9 (24.8)	11.1 (25.6)	12.3 (31.0)	11.4 (26.1)	11.2 (23.8)	9.5 (20.1)
	Median (Q1–Q3)	1 (0–11)	0 (0–11)	3 (0–11)	1 (0–11)	1 (0–12)	1 (0–11)	0 (0–9)
Days spent in LTC	Mean (SD)	94.2 (149.5)	106.2 (156.8)	98.0 (158.0)	89.7 (143.5)	96.9 (150.9)	88.6 (148.0)	72.3 (133.8)
	Median (Q1–Q3)	0 (0–191)	0 (0–276)	0 (0–229)	0 (0–130)	0 (0–213)	0 (0–161)	0 (0–41)
Number of primary care visits in previous year	Mean (SD)	5.9 (6.4)	7.2 (9.0)	5.3 (6.9)	6.1 (6.5)	5.8 (6.4)	5.6 (5.5)	5.8 (5.2)
	Median (Q1–Q3)	4 (1–9)	5 (1–11)	3 (1–7)	5 (2–9)	4 (1–9)	4 (1–8)	4 (2–9)
Time from catheterization to index prescription (day)	Mean (SD)	37.2 (27.6)	35.9 (29.8)	37.1 (28.7)	38.6 (28.6)	36.8 (27.1)	38.2 (27.2)	34.9 (28.2)
	Median (Q1–Q3)	35 (11–60)	34 (4–61)	36 (9–64)	37 (10–64)	34 (12–58)	36 (14–60)	32 (8–58)
Antibiotic time relative to culture finalization	Before	524 (11.8%)	53 (17.1%)	15 (8.7%)	60 (10.4%)	297 (12.3%)	69 (9.1%)	30 (15.6%)
	After	3,912 (88.2%)	257 (82.9%)	157 (91.3%)	519 (89.6%)	2,127 (87.7%)	690 (90.9%)	162 (84.4%)
Organism	Non-*E. coli*	2,943 (66.3%)	151 (48.7%)	121 (70.3%)	374 (64.6%)	1,646 (67.9%)	511 (67.3%)	140 (72.9%)
	*E. coli*	1,493 (33.7%)	159 (51.3%)	51 (29.7%)	205 (35.4%)	778 (32.1%)	248 (32.7%)	52 (27.1%)
Number of urine culture in previous year	Mean (SD)	2.9 (3.3)	3.6 (4.5)	2.6 (3.0)	2.8 (3.2)	3.0 (3.2)	2.9 (3.5)	2.9 (3.0)
	Median (Q1–Q3)	2 (1–4)	2 (1–4)	1 (1–4)	2 (1–4)	2 (1–4)	2 (1–4)	2 (1–4)
Number of positive urine culture in previous year	Mean (SD)	1.2 (1.9)	1.5 (2.5)	0.8 (1.3)	1.0 (1.8)	1.2 (1.9)	1.1 (2.0)	1.2 (1.7)
	Median (Q1–Q3)	0 (0–2)	1 (0–2)	0 (0–1)	0 (0–1)	0 (0–2)	0 (0–2)	1 (0–2)
Number of antibiotic courses in previous year	Mean (SD)	4.1 (6.0)	4.1 (5.5)	3.1 (3.3)	3.2 (3.8)	4.5 (7.0)	3.7 (4.6)	4.8 (6.3)
	Median (Q1–Q3)	2 (1–5)	3 (1–5)	2 (1–5)	2 (1–5)	3 (1–5)	2 (1–5)	3 (1–6)

### Effectiveness of CA-UTI Treatment Regimens

Of 4,436 CA-UTI patients, 2,709 (61.1%) patients experienced the primary outcome of treatment failure within 60 days of antibiotic dispensing. Repeat urinary antibiotic prescription was the most common component outcome driving treatment failure, occurring in 2,514 (56.7%). All-cause hospitalization occurred in 306 (6.9%) and mortality in 223 (5.0%) patients (Supplementary Tables 1(a) and 1(b)).

After adjusting for covariates, when comparing to a reference of TMP-SMX, treatment with fluoroquinolones was associated with a 9% lower risk of treatment failure in CA-UTI (RR 0.91, 95% CI: 0.85–0.98). Other antibiotics were not associated with a statistically significant difference in risk for treatment failure: amoxicillin-clavulanate RR 1.08 (95% CI: 0.98–1.19); nitrofurantoin RR 1.02 (95% CI: 0.94–1.10); fosfomycin RR 1.04 (95% CI: 0.92–1.18) compared to TMP-SMX. Patients receiving very short courses (1–4 d) were more likely to experience treatment failure (RR 1.15, 95% CI: 1.05–1.27) compared to those receiving 5–7 days of therapy. The longest range of duration of therapy of 8–14 days was not associated with a statistically significantly better outcome (RR 1.05, 95% CI: 0.99–1.11) than treatment for 5–7 days (Table 3).

**Table 3.** Treatment Failure for CA-UTI Patients Based on Antibiotic Selection and Duration

	Unadjusted		Adjusted*	
Antibiotic selection	RR (95% CI)	*P*-value	RR (95% CI)	*P*-value
Amoxicillin-clavulanate	1.09 (0.99, 1.21)	0.08	1.08 (0.98, 1.19)	0.12
Amoxicillin	0.93 (0.83, 1.04)	0.21	0.94 (0.84, 1.05)	0.27
Cefadroxil, Cephalexin	1.09 (1.00, 1.19)	0.06	1.09 (1.00, 1.19)	0.05
Nitrofurantoin	0.99 (0.92, 1.07)	0.84	1.02 (0.94, 1.10)	0.65
Trimethoprim-sulfamethoxazole	referent	.	referent	.
Ciprofloxacin, Levofloxacin	0.91 (0.84, 0.98)	0.01	0.91 (0.85, 0.98)	0.02
Fosfomycin	1.05 (0.92, 1.19)	0.46	1.04 (0.92, 1.18)	0.51

Antibiotic duration	RR (95% CI)	*P*-value	RR (95% CI)	*P*-value
1–4 d	1.14 (1.03, 1.26)	0.01	1.15 (1.05, 1.27)	< 0.01
5–7 d	referent	.	referent	.
8–14 d	1.05 (0.99, 1.12)	0.08	1.05 (0.99, 1.11)	0.12

* Adjusted for age, sex, Charlson comorbidity score, acute care, and long-term care days in the past 12 months.

Patients residing in community at the time of the index prescription had a more pronounced reduction in treatment failure with fluoroquinolone use (community RR 0.89, 95% CI: 0.81–0.98; LTC RR 0.97, 95% CI: 0.87–1.09) and a more pronounced increase in treatment failure for ultra-short courses (community: RR 1.19, 95% CI: 1.06–1.34; LTC 1.04, 95% CI: 0.87–1.25). However, patients in LTC were more likely to experience treatment failure with first-generation cephalosporins (community RR: 1.05, 95%CI: 0.92 to 1.19; LTC RR: 1.15, 95% CI: 1.02–1.30) (Supplementary Table 2).

A positive urine culture for the same organism within 60 days of antibiotic prescription was more common in patients treated with fosfomycin RR 1.47 (95% CI: 1.13–1.91), amoxicillin-clavulanate RR 1.22 (95% CI 0.99–1.51) and nitrofurantoin RR 1.16 (95% CI: 0.99–1.36). On the other hand, fluoroquinolones were associated with a statistically significantly lower risk for positive culture with the same organism within 60d RR 0.74 (95% CI: 0.62–0.88).

Our findings were robust to two sensitivity analyses, showing similar estimates when excluding patients who had more than one antibiotic prescription within 3 days of positive urine culture, and when shortening the threshold for prior catheterization to 30 days.

### Safety of CA-UTI Treatment Regimens

Harms of antibiotic treatment regimens were infrequently detected. Overall, 44 (1.0%) were detected to have the composite outcome of antibiotic harm within 60 days of index prescription. Diarrhea (0.6%, n = 27), *C. difficile* (0.2%, n = 9), and general medication adverse events (0.2%, n = 9) were the most commonly captured adverse outcomes (Supplementary Tables 1(a) and 1(b)). No statistically significant differences were detected between different treatment regimens.

## Discussion

In non-hospitalized older adults with presumed CA-UTI, we identified differences in the effectiveness of antibiotic treatment regimens. Fluoroquinolones were associated with a marginally lower risk of treatment failure. Treatment regimens of ≤ 4 days were associated with worse outcomes compared to > 4 days of treatment. We did not identify significant differences for durations longer than 7 days, compared to 5–7 days, or between non-fluoroquinolone antibiotic classes.

Our findings echo those of previous studies indicating high rates of clinical cure when using fluoroquinolones for uncomplicated UTI. Some analyses have identified superiority of fluoroquinolones when compared to non-fluoroquinolones for UTI,^20,21^ perhaps due to their high urinary and tissue concentrations leading to eradication of pathogens both in the urinary tract as well as in vaginal and colonic flora. This concept is supported by our findings that fluoroquinolones were associated with a lower rate of repeat positive urine cultures after treatment. Further, *E. coli* represented only 1/3 of urinary organisms in our study, which may lend fluoroquinolones an advantage due to their broad spectrum of activity against non-*E. coli* pathogens such as *Proteus* spp., *Klebsiella* spp., and *Pseudomonas aeruginosa*. On the other hand, the primary outcome is driven by repeat antibiotic use, so reduction in treatment failure with fluoroquinolones may reflect less antibiotic use for asymptomatic bacteriuria, an indication for which antibiotics should not be prescribed in older adults.^22^ Regardless of the reason for the lower risk of treatment failure, any benefits of fluoroquinolone use must be weighed against their potential risks. Although our study was not powered to identify differences in adverse events, fluoroquinolones have been associated with rare but severe harms including collagen-associated toxicity,^23^ retinal detachment,^24^ peripheral neuropathy,^25^ and perturbations of gut microbiota including elevated risk of *C. difficile* infection.^26^ Additionally, population-level risks of high fluoroquinolone consumption and resistance to these agents have been established.^27^

Although previous studies have identified a potentially higher risk of treatment failure in patients with cystitis treated with fosfomycin^28^ and nitrofurantoin,^29^ we found similar effectiveness for these agents when compared to TMP-SMX. This may be due to differences in population or differences in definitions used, given our outcomes are based on administrative data and do not account for symptom resolution. Notably, fosfomycin and nitrofurantoin appear to be associated with a higher risk of positive urine culture within 60 days of treatment, which may not necessarily equate to repeat infection in the absence of symptoms.

Our findings suggest that 5–7 days is an appropriate duration of therapy for CA-UTI. This range of duration appears to be similarly effective to longer courses for CA-UTI aligns with most recent recommendations and supports avoidance of unnecessarily prolonged durations of therapy for this indication.^9,10^ However, courses ≤ 4 days appear to be less effective suggesting ultra-short courses may not be sufficient to eradicate pathogens in CA-UTI. Courses of antibiotic therapy for CA-UTI > 4 days in length may be needed due to a longer time to eradicate bacteria given the formation of biofilm.^30^ Alternatively, this could reflect lower efficacy with agents typically used in short courses (eg, fosfomycin).

This study benefits from a large sample of province-wide administrative data from almost 100 laboratories, thereby improving generalizability. Antibiotic use is based on dispensed data, as such duration is intention to treat, and limits the likelihood of immortal time bias. Due to dispensing practices in LTC, we allowed for two consecutive 7-day prescriptions with the same antibiotic to be considered a full 14-day course. Although this approach allows us to evaluate LTC patients, it could introduce bias as patients that failed prior to 7 days may not receive their second part of the prescription. However stratified results by setting do not illustrate any differences in outcomes between 5 and 7 days or 8 and 14 days for community compared to LTC. Given the observational nature of the data, there is the possibility for unmeasured confounding across treatment regimens. However, our adjusted estimates after accounting for age, sex, comorbidities, and healthcare exposure did not significantly alter the crude estimates for treatment failure, suggesting minimal confounding. Secondly, treatment failure was driven largely by repeat antibiotic use, and it was not possible to ascertain whether initial treatment or repeat antibiotic use was for symptomatic UTI, asymptomatic bacteriuria, or for an unrelated non-urinary infection as indications for prescribing are not available for all patients. Prescribing for asymptomatic bacteriuria is highly likely given the high percentage (88%) of antibiotics initiated after culture finalization. It was not possible to ascertain if catheter use was indwelling at the time of infection for all patients, but a sensitivity analysis evaluating catheterization closer to the index event showed similar findings. Future research should incorporate an assessment for symptomatology, which is not available in administrative datasets, given the potential harms associated with treating asymptomatic bacteriuria.^22^

This research generates hypotheses for future prospective trials of CA-UTI, including an evaluation of which populations can receive agents with lower collateral damage and whether longer courses of therapy mitigate potentially reduced effectiveness. This work highlights the challenges of achieving antimicrobial stewardship in patients with CA-UTI and reinforces the importance of preventing CA-UTI in the first place through appropriate catheter stewardship, diagnostic stewardship to reduce urine culturing in the absence of symptoms, and avoidance of treating asymptomatic bacteriuria.

In conclusion, there is an apparent difference in real-world effectiveness between antibiotic treatment regimens for CA-UTI. Although most treatment options for CA-UTI have a similar risk of treatment failure, fluoroquinolones, and treatment durations ≥ 5 days in duration appear to be associated with modestly improved clinical outcomes. However, any difference in effectiveness with fluoroquinolone use should be weighed against potential harms of therapy. From a duration of therapy perspective, this study provides reassurance that relatively short courses of 5–7 days may be reasonable for CA-UTI.

## Supplementary material

For supplementary material accompanying this paper visit http://doi.org/10.1017/ash.2023.176. click here to view supplementary material

## Data sharing

The dataset from this study is held securely in coded form at ICES. Although data-sharing agreements prohibit ICES from making the dataset publicly available, access may be granted to those who meet prespecified criteria for confidential access, available at www.ices.on.ca/ DAS. The full dataset creation plan and underlying analytic code are available from the authors on request, with the understanding that the programs may rely on coding templates or macros that are unique to ICES.
