# Supplementary material for: Antibiotic Selection and Duration for Catheter-Associated Urinary Tract Infection in Non-Hospitalized Older Adults: A Population-Based Cohort Study
Source: Antimicrob Steward Healthc Epidemiol. 2023 Aug 1;3(1):e132. doi: 10.1017/ash.2023.176 (PMC10428148; doi:10.1017/ash.2023.176)
Supplement: Supplementary file 1 [file S2732494X23001766sup001.docx]

**Supplementary Table 1a. Treatment Outcomes for CA-UTI Patients by Antibiotic Selection**

| **Characteristics** |  | **Antibiotic selection** | | | | | | |
| --- | --- | --- | --- | --- | --- | --- | --- | --- |
|  | **Total** | **Amoxicillin Ampicillin** | **Amoxicillin-clavulanate** | **Cefadroxil, Cephalexin** | **Nitrofurantoin** | **Trimethoprim-sulfamethoxazole** | **Ciprofloxacin, Levofloxacin** | **Fosfomycin** |
|  | **N=4,436** | **N=306** | **N=300** | **N=431** | **N=1,083** | **N=678** | **N=1,360** | **N=278** |
| **Composite of treatment failure within 60 days after index prescription** | 2,709 (61.1%) | 175 (57.2%) | 201 (67.0%) | 288 (66.8%) | 660 (60.9%) | 420 (61.9%) | 765 (56.3%) | 200 (71.9%) |
| Repeat urinary antibiotic | 2,514 (56.7%) | 165 (53.9%) | 185 (61.7%) | 268 (62.2%) | 614 (56.7%) | 384 (56.6%) | 714 (52.5%) | 184 (66.2%) |
| All-cause hospitalization | 306 (6.9%) | 15 (4.9%) | 16 (5.3%) | 28 (6.5%) | 77 (7.1%) | 41 (6.0%) | 108 (7.9%) | 21 (7.6%) |
| Positive blood culture with same organism | 9 (0.2%) | - | 0 (0.0%) | 0 (0.0%) | - | - | - | 0 (0.0%) |
| All-cause mortality | 223 (5.0%) | 19 (6.2%) | 26 (8.7%) | 26 (6.0%) | 44 (4.1%) | 33 (4.9%) | 59 (4.3%) | 16 (5.8%) |
| **Positive urine culture with same organism within 60 days after index prescription** | 1,144 (25.8%) | 68 (22.2%) | 95 (31.7%) | 125 (29.0%) | 320 (29.5%) | 174 (25.7%) | 256 (18.8%) | 106 (38.1%) |
| **Composite of cause-specific hospitalization or ED visits within 60 days after index prescription** | 358 (8.1%) | 21 (6.9%) | 25 (8.3%) | 38 (8.8%) | 77 (7.1%) | 55 (8.1%) | 112 (8.2%) | 30 (10.8%) |
| UTI | 355 (8.0%) | 20 (6.5%) | 25 (8.3%) | 38 (8.8%) | 75 (6.9%) | 55 (8.1%) | 112 (8.2%) | 30 (10.8%) |
| Pyelonephritis | - | 0 (0.0%) | 0 (0.0%) | 0 (0.0%) | - | 0 (0.0%) | 0 (0.0%) | 0 (0.0%) |
| Sepsis | 27 (0.6%) | - | - | - | 7 (0.6%) | - | 8 (0.6%) | - |
| **Composite of any antibiotic harms within 60 days after index prescription** | 44 (1.0%) | 6 (2.0%) | - | - | 9 (0.8%) | 6 (0.9%) | 18 (1.3%) | - |
| *C. difficile* | 9 (0.2%) | - | 0 (0.0%) | 0 (0.0%) | - | - | - | - |
| Diarrhea | 27 (0.6%) | - | - | 0 (0.0%) | 6 (0.6%) | - | 11 (0.8%) | - |
| Antibiotic allergy | - | 0 (0.0%) | 0 (0.0%) | 0 (0.0%) | - | 0 (0.0%) | 0 (0.0%) | 0 (0.0%) |
| General medication adverse events | 9 (0.2%) | 0 (0.0%) | 0 (0.0%) | - | - | - | - | - |

Dashes (-) indicate insufficient data due to small cell size of <6 patients

**Supplementary Table 1b. Treatment Outcomes for CA-UTI Patients by Antibiotic Duration**

| **Characteristics** |  | **Antibiotic duration** | | |
| --- | --- | --- | --- | --- |
|  | **Total** | **1-4 days** | **5-7 days** | **8-14 days** |
|  | N=4,436 | N=482 | N=3,003 | N=951 |
| **Composite of treatment failure within 60 days after index prescription** | 2,709 (61.1%) | 335 (69.5%) | 1,784 (59.4%) | 590 (62.0%) |
| Repeat urinary antibiotic | 2,514 (56.7%) | 314 (65.1%) | 1,658 (55.2%) | 542 (57.0%) |
| All-cause hospitalization | 306 (6.9%) | 38 (7.9%) | 199 (6.6%) | 69 (7.3%) |
| Positive blood culture with same organism | 9 (0.2%) | 0 (0.0%) | - | - |
| All-cause mortality | 223 (5.0%) | 22 (4.6%) | 145 (4.8%) | 56 (5.9%) |
| **Positive urine culture with same organism within 60 days after index prescription** | 1,144 (25.8%) | 154 (32.0%) | 767 (25.5%) | 223 (23.4%) |
| **Composite of cause-specific hospitalization or ED visits within 60 days after index prescription** | 358 (8.1%) | 37 (7.7%) | 246 (8.2%) | 75 (7.9%) |
| UTI | 355 (8.0%) | 37 (7.7%) | 243 (8.1%) | 75 (7.9%) |
| Pyelonephritis | - | 0 (0.0%) | - | 0 (0.0%) |
| Sepsis | 27 (0.6%) | - | 18 (0.6%) | - |
| **Composite of any antibiotic harms within 60 days after index prescription** | 44 (1.0%) | 7 (1.5%) | 30 (1.0%) | 7 (0.7%) |
| *C. difficile* | 9 (0.2%) | - | 6 (0.2%) | - |
| Diarrhea | 27 (0.6%) | - | 17 (0.6%) | - |
| Antibiotic allergy | - | 0 (0.0%) | - | 0 (0.0%) |
| General medication adverse events | 9 (0.2%) | - | 7 (0.2%) | - |

Dashes (-) indicate insufficient data due to small cell size of <8 patients

**Supplementary Table 2.** **Treatment Failure for CA-UTI Patients Based on Antibiotic Selection and Duration Stratified by Healthcare Setting**

|  | **Unadjusted** | |  | **Adjusted*** | |
| --- | --- | --- | --- | --- | --- |
|  | **Community**  **RR (95%CI)** | **LTC**  **RR (95%CI)** |  | **Community**  **RR (95%CI)** | **LTC**  **RR (95%CI)** |
| Antibiotic Selection |  |  |  |  |  |
| Amoxicillin-clavulanate | 1.10 (0.94, 1.28) | 1.08 (0.94, 1.23) |  | 1.09 (0.94, 1.27) | 1.08 (0.94, 1.23) |
| Amoxicillin/Ampicillin | 0.97 (0.83, 1.14) | 0.89 (0.76, 1.05) |  | 0.98 (0.84, 1.15) | 0.91 (0.77, 1.07) |
| Cefadroxil, Cephalexin | 1.05 (0.93, 1.20) | 1.13 (1.00, 1.28) |  | 1.05 (0.92, 1.19) | 1.15 (1.02, 1.30) |
| Nitrofurantoin | 0.99 (0.89, 1.09) | 1.03 (0.92, 1.16) |  | 1.00 (0.91, 1.11) | 1.05 (0.93, 1.19) |
| Trimethoprim-sulfamethoxazole | referent | referent |  | referent | referent |
| Ciprofloxacin, Levofloxacin | 0.89 (0.81, 0.99) | 0.96 (0.86, 1.08) |  | 0.89 (0.81, 0.98) | 0.97 (0.87, 1.09) |
| Fosfomycin | 1.06 (0.90, 1.24) | 1.09 (0.88, 1.36) |  | 1.07 (0.92, 1.26) | 1.11 (0.89, 1.38) |
| Antibiotic Duration |  |  |  |  |  |
| 1-4 days | 1.20 (1.07, 1.35) | 1.03 (0.86, 1.23) |  | 1.19 (1.06, 1.34) | 1.04 (0.87, 1.25) |
| 5-7 days | referent | referent |  | referent | referent |
| 8-14 days | 1.05 (0.97, 1.13) | 1.07 (0.98, 1.16) |  | 1.04 (0.96, 1.12) | 1.05 (0.97, 1.15) |

* Adjusted for age, sex, Charlson comorbidity score, acute care and long-term care days in the past 12 months

**Supplement: Definitions**

*Definition of Urinary Catheterization*

- - 1. RAI-HC: I2b Indwelling Urinary Catheter OR InterRAI-HC: H2 Urinary Collection Device [Excludes pads/briefs]
    2. CCRS: H3D Indwelling Urinary Catheter
    3. OHIP: Z602, Z603, Z611 (urinary catheterization)

*Definition of Complicated or Upper Urinary Tract Infection (for Exclusion) ICD-10 codes*

N10 (acute pyelitis, pyelonephritis)

N11.0, (chronic nonobstructive reflux-associated pyelonephritis)

N11.1 (chronic obstructive pyelonephritis)

N13.6 (pyonephrosis)

N15.1 (renal and perinephric abscess),

N34.0 (urethral abscess)

N41.0, N41.1, N41.2, N41.3 (acute and chronic prostatitis, prostate abscess, prostatocystitis)

N43.1 (infected hydrocele)

N45.0,

N45.9 (orchitis/epididymitis with or without abscess)
